# A reference consensus genetic map for molecular markers and economically important traits in faba bean (*Vicia faba* L.)

**DOI:** 10.1186/1471-2164-14-932

**Published:** 2013-12-30

**Authors:** Zlatko Satovic, Carmen M Avila, Serafin Cruz-Izquierdo, Ramón Díaz-Ruíz, Gloria M García-Ruíz, Carmen Palomino, Natalia Gutiérrez, Stefania Vitale, Sara Ocaña-Moral, María Victoria Gutiérrez, José I Cubero, Ana M Torres

**Affiliations:** 1 IFAPA, Centro Alameda del Obispo, Área de Mejora y Biotecnología, Avda. Menéndez Pidal s/n, Apdo. 3092, Córdoba 14080, Spain; 2 Departamento de Mejora Genética, IAS-CSIC, Apdo. 4084, Córdoba 14080, Spain; 3 Present addresses: Department of Seed Science and Technology, Faculty of Agriculture, University of Zagreb, Zagreb, Croatia; 4 Colegio de Postgraduados, Recursos Genéticos y Productividad – Genética, Campus Montecillo, Km 36.5 Carretera México-Texcoco, C.P., Texcoco, Edo. de México 56230, México; 5 Colegio de Postgraduados, Campus Puebla, Km 125.5 Carretera México-Puebla, C.P., Puebla, Pue 72760, México

**Keywords:** Faba bean, Consensus map, Gene based markers, Quantitative trait loci, Comparative mapping, Molecular breeding, Marker-assisted selection, Genomics

## Abstract

**Background:**

Faba bean (*Vicia faba* L.) is among the earliest domesticated crops from the Near East. Today this legume is a key protein feed and food worldwide and continues to serve an important role in culinary traditions throughout Middle East, Mediterranean region, China and Ethiopia. Adapted to a wide range of soil types, the main faba bean breeding objectives are to improve yield, resistance to biotic and abiotic stresses, seed quality and other agronomic traits. Genomic approaches aimed at enhancing faba bean breeding programs require high-quality genetic linkage maps to facilitate quantitative trait locus analysis and gene tagging for use in a marker-assisted selection. The objective of this study was to construct a reference consensus map in faba bean by joining the information from the most relevant maps reported so far in this crop.

**Results:**

A combination of two approaches, increasing the number of anchor loci in diverse mapping populations and joining the corresponding genetic maps, was used to develop a reference consensus map in faba bean. The map was constructed from three main recombinant inbreed populations derived from four parental lines, incorporates 729 markers and is based on 69 common loci. It spans 4,602 cM with a range from 323 to 1041 loci in six main linkage groups or chromosomes, and an average marker density of one locus every 6 cM. Locus order is generally well maintained between the consensus map and the individual maps.

**Conclusion:**

We have constructed a reliable and fairly dense consensus genetic linkage map that will serve as a basis for genomic approaches in faba bean research and breeding. The core map contains a larger number of markers than any previous individual map, covers existing gaps and achieves a wider coverage of the large faba bean genome as a whole. This tool can be used as a reference resource for studies in different genetic backgrounds, and provides a framework for transferring genetic information when using different marker technologies. Combined with syntenic approaches, the consensus map will increase marker density in selected genomic regions and will be useful for future faba bean molecular breeding applications.

## Background

Faba bean (*Vicia faba* L.) is an important food and fodder crop worldwide and a staple in Middle East, Central and East Asia and North Africa. In terms of cultivation area, faba bean ranks fourth among the cool-season food legumes (close to 2.5 million hectares per year) after chickpea, pea and lentil (http://faostat.fao.org). Its agricultural role is currently increasing as the crop is receiving a renovated interest in European countries, North America and Australia.

Faba bean is a diploid legume crop (2n = 2× = 12) with facultative cross-pollination and has one of the largest described genomes among legumes. The genome size is ~13,000 Mb, more than 25 times larger than that of the model *Medicago truncatula,* and contains more than 85% of repetitive DNA [1]. The large chromosome size has allowed faba bean to become one of the cytogenetically best characterised plant species. However, it has also difficulted the development of saturated linkage maps and the identification of relevant genes/QTLs (Quantitative Trait Loci). Moreover, it precludes whole-genome shotgun assembly with next generation sequencing technologies.

Genetic linkage maps are essential tools for a wide range of genetic and breeding applications, including the study of inheritance of qualitative and quantitative traits and the identification of markers linked to relevant agronomic traits. The availability of high-density maps enhances the breeding process through the application of association analyses, map-based cloning or marker-assisted approaches. Table 1 presents a summary of the faba bean linkage maps reported so far.

**Table 1:** Information of relevant faba bean mapping populations highlighting the ones used to construct this composite map

**Reference**	**Cross**	**Mapping population**	**No. individuals**	**No. markers**	**No. LGs**^**a**^	**Length (cM)**	**Uses**^**b**^
Van de Ven et al. [2]		BC		17	7 (-)	231	
Torres et al. [3]		2 F_2_	20	51	11 (1)	~300	
Ramsay et al. [4]		BC		23	7 (-)	~300	
**Satovic et al. **[5]	c	7 F_2_	813	157	48 (6)	~850	T/C
**Vaz Patto et al. **[6]	Vf6 × Vf27	3 F_2_	175	116	13 (7)	~1200	T/C
**Román et al. **[7]**,**[8]	Vf6 × Vf136	F_2_	196	121	16 (9)	1446	Q
**Román et al. **[9]	d	11 F_2_	654	192	14 (5)	1559	T/C
**Ávila et al. **[10]	29H × Vf136	F_2_	159	103	18 (6)	1308	Q
**Ellwood et al. **[11]	Vf6 × Vf27	RIL	96	135	12 (-)	1686	
Arbaoui et al. [12]	Côte d’Or × BPL14628	RIL	101	131	21 (-)	~980	Q
**Díaz et al. **[17]**,**[19]	Vf6 × Vf136	RIL	165	277	21 (9)	2857	Q
**Cruz-Izquierdo et al. **[13]	Vf6 × Vf27	RIL	124	258	16 (8)	1874	Q
Ma et al. [14]	91825 × K1563	F_2_	129	128	15 (-)	1587	
**Gutiérrez et al. (in press) **[15]	29H × Vf136	RIL	119	171	29 (15)	1402	Q
**This study**	e	3 RIL	408	587^f^	6 (6)	3515	C
				151^g^	37 (7)	1171	C

^a^Between brackets no. of linkage groups (LGs) assigned to chromosomes.

^b^T: Assignation of linkage groups to chromosomes by trisomic segregation; C: Development of a composite map; Q: QTL analysis.

^c^Vf6 × Vf2; Vf6 × Vf33; Vf6 × Vf159.

^d^Vf6 × Vf2; Vf6 × Vf27; Vf6 × Vf33; Vf6 × Vf136; Vf6 × Vf159.

^e^Vf6 × Vf27; Vf6 × Vf136; 29H × Vf136.

^f^Data of the six main LGs adscribed to chromosomes.

^g^Data of the minor LGs.

Studies carried out by the IFAPA group and considered in this study in bold.

Prior to 1990, only a few morphological and isozyme loci were mapped in the *V. faba* genome and no extended linkage groups (LGs) had been reported. Primary trisomics and translocation stocks allowed the first assignment of genes and LGs to specific chromosomes [3,16-19]. This approach was further explored to develop physically localized markers and microsatellites (or SSR-Simple Sequence Repeats) from specific chromosomic regions [20]. This led to the integration of the first genetic and physical maps and allowed the unambiguous assignation of LGs to their respective chromosomes.

Preliminary maps constructed with F_2_ populations were mostly based on dominant markers such as RAPDs together with morphological, isozyme, seed storage protein genes and microsatellites, which saturated different areas of the genome [5-8,21,22]. First attempts to map genes/QTLs for seed weight [6] and resistance to a parasitic plant (*Orobanche crenata*) and fungal diseases (*Ascochyta fabae* and *Uromyces viciae-fabae*) were reported [7,8,10,23]. Using a F_2_ population from the cross Vf6 × Vf136, a linkage map was developed to locate QTLs controlling crenate broomrape (*O. crenata*) [7] and *A. fabae* resistance [8]. Nine of the 16 LGs reported could be assigned to specific chromosomes thanks to markers that were common with those of previous studies. Subsequently, a linkage map of an F_2_ population from the cross 29H × Vf136, segregating for resistance to the two pathogens, was constructed in which 6 of the 18 LGs were assigned to chromosomes [10].

These faba bean maps did not allow wider mapping comparisons, since they mostly shared dominant and anonymous markers such as RAPDs, with scarce transferrability between genotypes and legume species. Despite this limitation, marker data of 11 F_2_ populations (Table 1), all sharing the common female parent Vf6, were used to construct a composite linkage map [9]. After joint segregation analysis of 501 markers in 654 individuals, 192 markers were included in 14 major LGs, of which 5 were unambiguously assigned to specific chromosomes (Table 1). This composite map covered 1,559 cM and was one of the most comprehensive faba bean genetic map published to date [9].

These maps with dominant markers in F_2_ were followed by more precise maps constructed in the corresponding RIL populations, using co-dominant markers. In addition to microsatellites, expressed sequence tags (EST) from other legume species emerged as efficient tools in faba bean. A large number of intron-targeted primer pairs (ITAPs), developed within the Grain Legumes Integrated Project-GLIP (http://www.pcgin.org/GLIP/pubrep.pdf), was tested and mapped in two faba bean inbred populations (Vf6 × Vf136, 29H × Vf136). These were used to validate QTLs underlying broomrape and *Ascochyta* resistance in different environments and genetic backgrounds [15,24-26]. A third RIL population derived from cross Vf6 × Vf27 was used to construct the first exclusively gene-based genetic map in faba bean. It contained 135 ITAPs joined in 12 unassigned LGs, that spanned 1,685 cM, and allowed for the first time the study of macrosyntenic relationships between *V. faba*, *M. truncatula*, *Lens culinaris* and other legume species [11]. After further saturation, the map was used to identify and validate QTLs controlling flowering time and other yield-related traits [13]. Recently, a new map was reported based on the F_2_ population from the cross 91825 × K1563, which includes 128 SSRs markers arranged in 15 unassigned LGs [14]. Unfortunately the lack of common markers prevented comparisons with previous mapping studies.

To date 14 major genetic maps have been constructed in faba bean (Table 1). Integrating the information of multiple populations from diverse genetic backgrounds offers several advantages over individual genetic maps: (i) a larger number of loci is mapped than in single crosses, (ii) the relative position of common markers can be determined across the mapping populations, (iii) better genome coverage and opportunities to validate marker order, (iv) better assignment of LGs to chromosomes, (v) it allows comparison of genes/QTLs of interest across maps and, (vi) it provides the basis for comparing genomes between related species [27-29]. Consensus genetic maps have been developed in many crops such as wheat [30], maize [31], barley [32] and rice [33], and in the legume crops soybean [34], pea [35], chickpea [36], phaseolus [37], pigeonpea [38], cowpea [39], groundnut [40] and red clover [41].

With the development of genome sequencing projects and expression studies in different model and crop legumes, the construction of a faba bean consensus genetic map has become possible. The objectives of this study were to: (1) saturate the faba bean maps developed in RIL progenies with common gene based markers to facilitate anchoring of linkage groups from different populations, (2) update the position of the most relevant faba bean QTLs controlling resistance and yield related traits using Bulked Segregant Analysis (BSA), and (3) construct a reference map integrating all the genomic information reported so far in this crop. To this aim, we fused information of 11 F_2_ populations and marker data of three RIL genetic maps to derive a consensus map including 729 markers and covering 4,602 cM. The six main LGs could be unambiguously assigned to their corresponding faba bean chromosomes. The map represents a significant improvement over single-population genetic maps and provides a new tool of reference for faba bean breeding and genomic approaches.

## Results

### Individual maps and QTL analysis

#### *Cross Vf6 × Vf27 (RIL1)*

The first RIL1 map [13] included 258 markers joined in 16 LGs and covering 1,875 cM. The linkage groups were composed of 2–45 loci with an average marker interval of 7.3 cM. The map allowed to identify and validate QTLs controlling 5 flowering and reproductive traits [13]: days to flowering (DF), flowering length (FL), pod length (PL), number of seeds per pod (NSP) and number of ovules per pod (NOP), located mainly in chromosomes (chr.) V and VI (Additional file 1: Table S1).

For the extended RIL1 map constructed herein, 313 polymophic markers were used in the global analysis (Table 2). Of these, 273 were assembled in 19 LGs, 11 of which could be assigned to specific chromosomes. The distance covered by the map was 2,183 cM with an average marker interval of 10 cM. Sixty five of the markers are common with the other two RIL populations, 25 with RIL2 (Vf6 × Vf136) only, 15 with RIL3 (29H × Vf136) only and 25 with both (Table 3).

**Table 2:** Number and type of markers genotyped in each inbred population

**Type of marker**		**RIL population**	
	**Vf6 × Vf27**	**Vf6 × Vf136**	**29H × Vf136**
ITAP	176	59	46
RAPD	107	327	145
SSR	16	6	6
Isozyme	5	4	0
RGA	4	5	5
Morphological trait	2	0	0
Seed storage protein	2	2	2
DR	1	1	1
Total	313	404	205

**Table 3:** Common markers used as anchors for map integration

**No.**	**Marker**	**Marker type**	**RIL population**		
			**Vf6 × Vf27**	**Vf6 × Vf136**	**29H × Vf136**
1	PeaβGlu	DR	x	x	
2	Prx-1	Isozyme	x	x	
3	Sod-1	Isozyme	x	x	
4	1433P	ITAP	x		x
5	6DCS	ITAP	x	x	x
6	AIGPb	ITAP	x	x	
7	AnMtS13	ITAP	x	x	x
8	AnMtS37	ITAP	x	x	
9	BGAL	ITAP	x		x
10	cgP137F	ITAP	x		x
11	GBNP	ITAP	x		x
12	GLIP171b	ITAP	x	x	
13	GLIP651	ITAP	x	x	
14	HBP2	ITAP	x		x
15	LG007	ITAP	x		x
16	LG031	ITAP	x	x	x
17	LG041	ITAP	x	x	
18	LG054	ITAP	x	x	
19	LG068	ITAP	x	x	x
20	Lup066	ITAP	x	x	x
21	Lup299	ITAP	x	x	x
22	MMK1a	ITAP	x		x
23	mtmt_GEN_00012_03_1	ITAP	x	x	x
24	mtmt_GEN_00022_02_1	ITAP		x	x
25	mtmt_GEN_00024_04_1	ITAP	x	x	
26	mtmt_GEN_00032_01_1/a	ITAP	x	x	
27	mtmt_GEN_00036_02_1/a	ITAP	x	x	x
28	mtmt_GEN_00103_01_1	ITAP	x	x	
29	mtmt_GEN_00447_04_3	ITAP	x		x
30	mtmt_GEN_00477_04_1	ITAP	x		x
31	mtmt_GEN_00510_01_1	ITAP	x		x
32	mtmt_GEN_00757_03_1	ITAP	x	x	x
33	mtmt_GEN_00861_03_1	ITAP	x		x
34	mtmt_GEN_00866_02_1	ITAP		x	x
35	mtmt_GEN_00892_01_3	ITAP	x	x	x
36	mtmt_GEN_00995_01_1	ITAP	x	x	x
37	mtmt_GEN_01017_03_3	ITAP	x	x	
38	mtmt_GEN_01102_02_1	ITAP	x	x	x
39	mtmt_GEN_01109_01_1	ITAP	x	x	x
40	mtmt_GEN_01115_02_1	ITAP	x		x
41	mtmt_GEN_01130_02_1	ITAP	x	x	x
42	mtmt_GEN_01951_11_1a	ITAP	x	x	x
43	Pis_GEN_14_7_1	ITAP		x	x
44	Pis_GEN_20_1_1	ITAP	x	x	
45	Pis_GEN_23_5_6_1	ITAP	x	x	
46	Pis_GEN_25_2_3_1	ITAP	x	x	
47	Pis_GEN_5_4_5_1	ITAP	x	x	
48	Pis_GEN_57_1_2_1	ITAP	x	x	
49	Pis_GEN_6_3_1	ITAP	x	x	x
50	Pis_GEN_7_1_2_1	ITAP	x	x	
51	psat_EST_00180_01_2	ITAP	x	x	
52	psat_EST_00190_01_1	ITAP	x		x
53	PsMnSOD	ITAP		x	x
54	psmt_EST_00196_01_1	ITAP	x	x	
55	RBPC/O	ITAP	x		x
56	RNAR	ITAP	x		x
57	SAT	ITAP	x	x	
58	TBB2	ITAP	x	x	x
59	UNK28	ITAP	x	x	
60	RGA01	RGA	x	x	x
61	RGA03	RGA	x	x	x
62	RGA08	RGA	x	x	x
63	RGA09	RGA	x	x	x
64	B3	Seed storage protein	x	x	
65	B4	Seed storage protein	x	x	x
66	GA4	SSR	x	x	x
67	GAII30	SSR	x	x	
68	GAII59	SSR	x	x	x
69	JF1GA3	SSR	x	x	x
	Total number of common markers		65	54	44

#### *Cross VF6 × VF136 (RIL2)*

The previously published RIL2 map [24,25] was based on 277 marker loci assembled in 21 LGs (16 consisting of 3 or more markers) that span 2,857 cM with an average marker interval of 12.7 cM. In this population, 2 QTLs controlling ascochyta blight resistance (*Af1* and *Af2*) were identified on chr. III and chr. II [24]. In parallel studies, 2 QTLs (*Of1* and *Of2*) controlling *O. foetida* resistance and 4r QTLs controlling *O. crenata* resistance (*Oc2-Oc5*) were detected [25]. *Oc2* and *Oc3* were stable in at least two of the three environments, while *Oc4* and *Oc5* were only detected in one environment and thus appeared to be environment-dependent.

**Figure 1:**
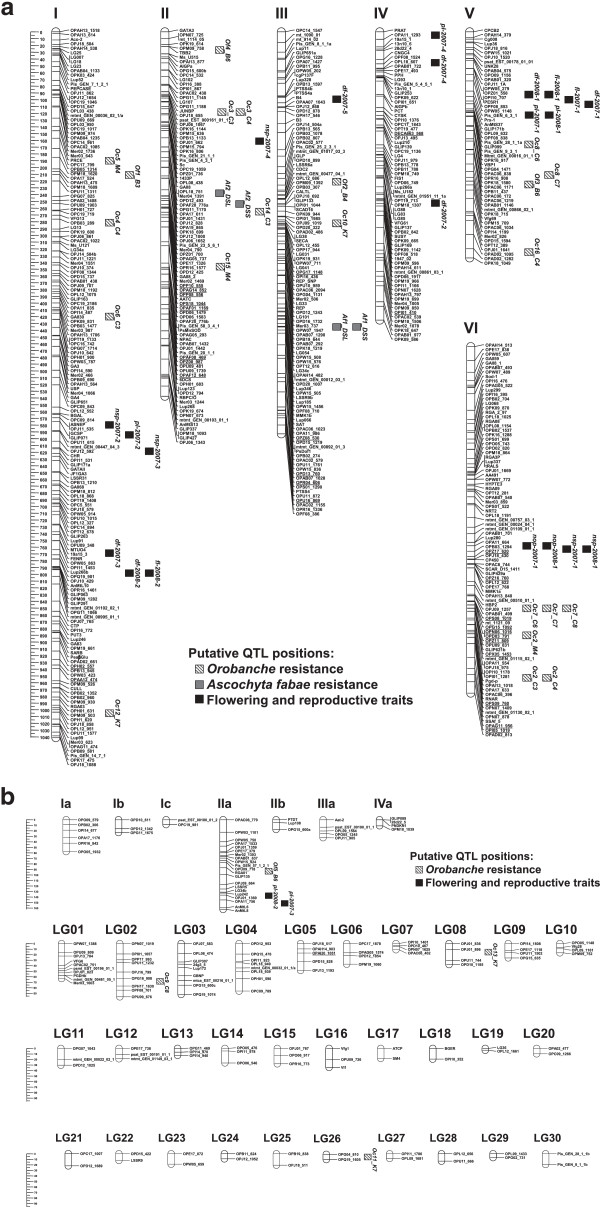
**Faba bean consensus map showing: (a) the six main linkage groups or chromosomes (578 loci) and (b) minor linkage groups (30) or small chromosome fragments (7).** Additional markers derived from the BSA analysis are underlined. Boxes represent putative locations of QTLs. Black boxes were used for flowering and yield related traits: days to flowering *(df*), flowering length (*fl*), pod length (*pl*), number of ovules per pod (*nop*) and number of seeds per pod (*nsp*). Grey boxes: *Ascochyta fabae* (*Af*) QTLs. Stripped boxes: *Orobanche crenata* (*Oc*) and *Orobanche foetida* QTLs. Marker distance is given in cM.

In an attempt to saturate the regions bearing the *O. crenata* and *A. fabae* QTLs, a BSA approach based on RAPD markers was applied. Two-hundred and eight of the 748 RAPD primers assayed in the cross revealed promising polymorphisms between at least one pair of bulks and were subsequently used to screen 14 plants individually. Only 41 of the RAPD primers maintained the expected pattern of polymorphism and were used to screen the entire RIL2 population, resolving 39 scorable polymorphic markers. Of these, 31 markers exhibited the expected segregation pattern and were thus included in the RIL data set for mapping and QTL analysis. Thirty of the 31 RAPD markers were mapped, 24 to target regions [11 to chr. VI (*Oc2*), 8 to chr. II (*Oc3* and *Af2*) and 5 to chr. III (*Af1*)] and 6 to other LGs (Table 4; Figure 1). To increase the number of common markers between different faba bean crosses, additional ITAP markers were scored and the map was reconstructed using 404 segregating loci.

**Table 4:** Linkage map of Vf6 × Vf136 (RIL2)

**No.**	Chromosome/LG*	**No. markers**	New markers**	**Length (cM)**	**Intermarker distance (cM)**
1	I-1	69	1 / 7	730.18	10.74
2	I-2	15	2 / 2	164.43	11.75
3	I-3	2	0 / 2	21.18	21.18
4	Ic	2	0 / 0	8.67	8.67
5	II	53	8 / 11	522.80	10.05
6	IIa	11	0 / 1	80.19	8.02
7	III-1	53	5 / 12	462.63	8.90
8	III-2	4	0 / 1	33.88	11.29
9	IV	38	2 / 2	346.53	9.37
10	V-1	27	0 / 5	290.67	11.18
11	V-2	7	0 / 1	55.24	9.21
12	VI-1	28	0 / 8	284.94	10.55
13	VI-2	24	11 / 3	228.73	9.94
14	LG01	8	0 / 0	61.63	8.80
15	LG04	6	0 / 0	86.05	17.21
16	LG05	5	1 / 0	48.55	12.14
17	LG06	4	0 / 0	37.40	12.47
18	LG11	2	0 / 0	20.27	20.27
19	LG21	2	0 / 0	22.14	22.14
20	LG22	2	0 / 2	11.93	11.93
21	LG23	2	0 / 0	18.80	18.80
	Mapped	364	30 / 57	3536.86	12.60
	Unmapped	40			
	Total	404			

*Linkage groups correspond to those of the composite map.

**Markers mapped in addition to already published linkage map [24,25]: first number represents the number of markers added by saturation mapping targeted at the regions conferring resistance to *O. crenata* and *Ascochyta fabae* (II, III-1, VI-2), while the second is the number of markers added to increase the number of common markers among different faba bean crosses.

The map obtained in this study consists of 364 mapped loci assembled into 21 LGs, of which 13 were assigned to specific chromosomes. The genome distance covered by the map is 3,537 cM, with an average density of one marker locus every 12.6 cM (Table 4). Fifty four markers included in RIL2 are common with the other two populations, 25 with RIL1 only, 4 with RIL3 only and 25 with both (Table 3).

As mentioned above, QTLs *Oc2* and *Oc3,* that confer broomrape resistance, were previously validated in 2 of the 3 field trials assayed [26]. By saturating the target regions we were able to identify significant QTLs in each trial. Thus, the conservation of QTLs both across generations and environments was confirmed. In case of *Oc2* (Additional file 1: Table S1B) a newly added marker, OPAG11_956_, was the closest to LOD peak value in both Córdoba 2003 and Córdoba 2004 datasets. Three additional QTLs were identified in chr. II (*Oc14_C3* and *Oc15_M4*) and V (*Oc15_C4*). None of these QTLs was stable in the different field assays or years, pointing towards an environment-dependent expression. In the case of ascochyta blight resistance, the analysis which was based on a more saturated map yielded higher LOD scores and narrower confidence intervals for both QTLs (*Af1* and *Af2*). The new marker OPZ08_530_ was the closest to LOD peak value of both *Af1* QTLs identified in leaves and stems (Additional file 1: Table S1B). Addition of new markers in target areas of the map was an efficient method to increase the genome coverage and to obtain more defined QTLs.

#### *Cross 29H × VF136 (RIL3)*

The third map used in the present study was reported recently and includes 172 markers spanning 1402 cM [15]. The linkage groups were composed of 2 to 25 loci with a marker interval of 9.87 cM. Seven QTLs for *O. crenata* (*Oc7* to *Oc13*) and 3 QTLs for *O. foetida* (*Of3* to *Of5*) were identified in this map. *Oc7* was detected along three years, explaining between 22% and 33% of the phenotypic variation. It has been suggested that *Oc2* (previously reported in RIL2) and *Oc7*, which are both located in chr. VI and validated in different environments and genetic backgrounds, might correspond to the same QTL region (Figure 1; Additional file 1: Table S1). The new analysis considered 205 marker loci segregating in this population, of which 25 were common to both other RIL populations, 25 to RIL1 only and 4 to RIL3 only (Table 3).

### Consensus linkage map

Three sets of faba bean mapping data were used in the construction of a consensus map connecting information of 11 F_2_ populations, marker data of 3 RILs, as well as new markers genotyped in the present study (Tables 1 and 2). The number of individual marker loci ranged from 313 in RIL1 to 404 in RIL2 and 205 in RIL3 (Table 2). Chi-square test was performed on new marker genotyping data for individual mapping population,s to test the null hypothesis of segregation ratios of 1:1. A variable percentage of distorted markers (P < 0.01) was observed, ranging from 2.93% in RIL3 to 7.35% in RIL1. A list of the marker loci is provided in Additional file 2: Table S2. QTL regions characterized in previous studies were also covered by the present consensus map.

The number of anchor markers for pairwise comparisons was initially small, with less than 20 markers in common. However, after the new marker analysis the number triplicated to 69 (2 isozymes, 4 SSRs, 2 legumines, 4 RGAs, 1 DR gene and 56 ITAPs). Sixty five of these, present in RIL1, were primarily used as bridges to integrate the individual maps into a single consensus map (Table 3). Twenty five markers were common in the 3 maps, while the remaining 44 were shared by at least 2 mapping populations. As a result, marker segregation data were assembled for a total of 828 marker loci, 759 of which were unique i.e. mapped only in one population (Table 5; Additional file 2: Table S2).

**Table 5:** **Composite map of faba bean (*Vicia faba *L.) genome**

**No.**	**Chromosome/LG**	**No. markers**	**Length (cM)**	**Intermarker distance (cM)**
1	I	165	1041.20	6.35
2	II	93	537.60	5.84
3	III	109	593.14	5.49
4	IV	70	425.20	6.16
5	V	53	333.60	6.42
6	VI	88	511.22	5.88
7	Ia	6	57.80	11.56
8	Ib	3	23.25	11.63
9	Ic	2	8.67	8.67
10	IIa	21	162.46	8.12
11	IIb	3	17.48	8.74
12	IIIa	5	32.63	8.16
13	IVa	4	11.02	3.67
14	LG01	10	63.25	7.03
15	LG02	9	96.45	12.06
16	LG03	9	91.99	11.50
17	LG04	8	86.04	12.29
18	LG05	5	48.55	12.14
19	LG06	4	37.40	12.47
20	LG07	4	11.07	3.69
21	LG08	4	37.86	12.62
22	LG09	4	25.98	8.66
23	LG10	4	16.75	5.58
24	LG11	3	30.36	15.18
25	LG12	3	18.75	9.38
26	LG13	3	12.63	6.31
27	LG14	3	26.43	13.22
28	LG15	3	28.03	14.01
29	LG16	3	34.35	17.18
30	LG17	2	18.24	18.24
31	LG18	2	19.48	19.48
32	LG19	2	3.48	3.48
33	LG20	2	9.80	9.80
34	LG21	2	22.14	22.14
35	LG22	2	11.93	11.93
36	LG23	2	18.80	18.80
37	LG24	2	9.34	9.34
38	LG25	2	20.67	20.67
39	LG26	2	9.37	9.37
40	LG27	2	8.41	8.41
41	LG28	2	13.43	13.43
42	LG29	2	6.89	6.89
43	LG30	2	19.37	19.37
	Mapped	729	4612.52	10.73
	Unmapped	99		
	Total	828		
	Main LGs (No. 1–6)	578	3441.96	6.02

A total of 729 marker loci were assembled into the 43 LGs constituting the consensus map (Figure 1; Table 5), while 99 markers remained unlinked. The 6 major LGs contained between 53 (chr. V) and 165 marker loci (chr. I), and were assigned to the corresponding chromosomes. Seven additional LGs (Ia to IVa) could also be assigned thanks to the presence of loci previously located in individual chromosomes. Thirty one LGs consisted of 2–5 markers, and the remaining 6 LGs contained between 6 and 21 loci (Table 5; Additional file 2: Table S2). The total length of the consensus genetic linkage map was 4,613 cM, of which 3,442 cM were covered by the 6 main LGs/chromosomes. The length of these major LGs ranged from 323 cM (chr. V) to 1041 cM (the large metacentric chr. I). The entire consensus map had an average marker density of one marker per 10.7 cM, which was reduced to 6 cM when considering only the 6 main LGs. The marker order of the integrated map was largely collinear with the three individual maps, although a few local inversions and marker rearrangements over short intervals were observed.

### Integration of QTL information

The number of QTL studies in faba bean is relatively low compared to other major legume species. Most traits have been genetically characterized in only one or two different mapping experiments, which limits the meta-analysis of QTLs in this species. Moreover, QTL intervals did not always include the minimum of two anchor markers, which is required for their projection onto the consensus map. Nevertheless, by comparing the maps published to date we provide a synthetic view of the most relevant loci controlling polygenic traits in faba bean. Further mapping of common markers between maps will be crucial to enhance the comparison of QTL positions from different mapping studies and to refine the localization of hot-spot genomic regions.

The 5 faba bean mapping experiments in the 3 RIL populations reported so far, identified 37 QTLs for 9 traits (Additional file 1: Table S1). Most of the QTL analyses focused on biotic stresses (e.g. broomrape and ascochyta blight resistance). The number of QTLs for broomrape resistance is 15 for *O. crenata* and 5 for *O. foetida*. Meanwhile the *A. fabae* resistance QTLs were reduced to 2, *Af1* and *Af2*, that were conserved among populations (F_2_ and RIL) and environments. Regarding flowering and yield related traits, the number of stable QTLs reported were 1 (NOP), 2 (FL), 4 (PL), 3 (NSP) and 5 (DF) (Additional file 1: Table S1). The distribution of these 37 QTLs varied from 9 in chr. I, 8 in chr. II, 4 in chr. III and VI, 3 in chr. IV, 6 in chr. V. The last three QTLs remained unassigned.

## Discussion

During the last decade, significant progress was made in the development of genotyping tools. This allowed the addition of a large number of robust and transferrable marker loci in the genetic maps of relevant crop species. In faba bean, mapping studies were initiated in the 90's with the development of the first maps in F_2_ populations using mostly RAPDs together with SSRs, isozymes and morphological markers. Previous studies comparing these linkage maps have been reported [9]. The use of a recurrent parent (Vf6) in all the F_2_ populations then allowed to join data from different progenies by means of common markers in the female parent. Moreover, the use of trisomic families for chromosomes III, IV, V and VI allowed allocation of LGs to chromosomes [9]. Ever since, attempts have been made to increase marker density using new SSRs and gene-based markers in RIL populations. The main objective of the present study was the development of a high density consensus genetic map that integrates all the relevant maps reported so far and serves as a reference map for the international faba bean community.

Building a consensus map is not possible without common or bridge loci on each LG or chromosome. For this reason, a number of additional markers was genotyped in each mapping population to increase the number of common markers among them. A bridge marker was considered as such when its name and position were the same in the different mapping populations. The genetic map was created combining two approaches: (a) increasing the number of anchor marker loci in the different populations, (b) merging the resulting genetic maps through markers common to three RIL populations with MergeMap [42] as reported in many other crop species [39,43-47].

Using this approach, segregation data for 729 marker were assembled on 43 LGs. In terms of marker order, the consensus map contains few changes compared to the individual maps. Small discrepancies in the marker order or position in some LGs might be due to (i) different population sizes used, (ii) weak linkages existing in the different maps, or (iii) missing or poor quality data, rather than to real chromosome rearrangements. As reported in previous studies in *Vitis vinifera* L. [47], phaseolus [37] or *Brassica napus *[44], the faba bean consensus marker order is significantly more reliable than that of the individual maps because of the higher number of individuals and recombination events occuring across three or more populations.

Based on previous LG/marker allocations, 13 of the larger LGs could be assigned to specific chromosomes while 30 LGs remained unassigned. Considering the enormous size of the faba bean genome, unassigned LGs may be due to recombination gaps at the distal ends of the main LGs because of a lack of marker loci. None of the main LGs differed considerably in marker density. The length of our core map was 3,431 cM, which is higher than the single RIL maps. In many other species the increased size of the composite map was attributed to an improved coverage of the chromosome ends [37,48-50].

**Figure 2:**
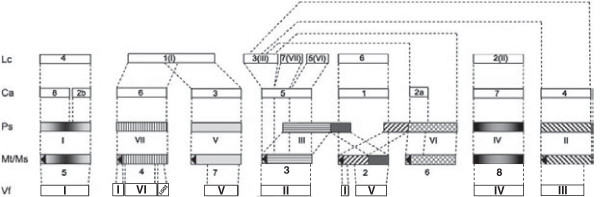
**Schematic representation of large-scale synteny blocks between chromosomes and chromosome segments of *M. truncatula *(Mt) and main cool season grain legumes (Source **[52]**, with modifications).** Chickpea (*Cicer arietinum*; Ca), faba bean (*Vicia faba*; Vf), lens (*Lens culinaris*; Lc), and pea (*Pisum sativum*; Ps). Bars representing *Medicago* and pea homologous chromosomal regions are shown with the same gray intensity or pattern. Arrows in the boxes indicate the orientation of the chromosomes (short arm - long arm) in the case of *Medicago*. The corresponding synteny blocks of faba bean, chickpea and lentil are represented by blank bars. The bars do not reflect the relative sizes of chromosome or chromosome segments and the break points of chromosomes are indicated approximately. The figure integrates data from [11,13,53-58] and this study.

All the individual maps reported to date allocate LGs to 5 of the 6 faba bean chromosomes, excluding chr. IV. After acknowledging the erroneous assignment of LG I.B, which actually corresponds to chr. IV [51], the new integrated map anchors for the first time the main LGs to the whole chromosome complement of the species. This information was used here to update the reported large-scale synteny between LGs and/or chromosomes of *M. truncatula* and cool season grain legumes such as pea, chickpea, lens and faba bean [52]. Figure 2 shows the main syntenic blocks and rearrangements among these species and their correspondence to the six faba bean chromosomes.

The integration of a high number of coding regions in the consensus map provides an excellent framework for downstream analyses, including comparisons between the locations of major genes for important traits or QTL positions between populations from different crosses. Moreover, combining syntenic studies with a consensus map will contribute to increase marker density in genomic regions of interest for indirect selection or for map based cloning [37]. Successful application of consensus maps for synteny based candidate gene identification and/or definition of QTL location has been extensively used both in cereals [59-62] and in legumes [35,37,63,64].

The RIL mapping populations considered in the consensus map were used previously for detecting QTLs of agronomically important traits. These are displayed in Figure 1, together with the QTLs identified in the improved Vf6 × Vf136 map and detected in the present study. In order to increase the density of loci around the OTLs for broomrape and ascochyta blight resistance, we used BSA in contrasted DNA pools. The BSA approach has been applied in numerous studies and provides a platform for high-resolution genetic analysis [65]. In the present study, 24 of 31 RAPD markers were mapped to the major linkage groups and allowed more accurate determination of QTL locations and effects. These results highlight the usefulness of BSA based on markers flanking QTLs, as an efficient tool for saturation of targeted regions, opening the possibility of future marker-assisted selection for these traits.

Faba bean has been considered a “genomic orphan” crop with a huge and complex genome and limited availability of genetic and genomic resources. At present, the situation has greatly improved thanks to the technological advances in high-throughput sequencing and genotyping, together with the access to genomic and transcriptomic tools. Genome-wide transcription profiling by deepSuperSAGE was recently used for quantifying the transcriptional changes elicited by *A. fabae* and to identify candidate resistance genes governing faba bean responses to this fungal pathogen [66]. Several genome libraries have been constructed and characterized for putative SSR sequences using the Roche 454 GS FLX Titanium Sequencing Platform [67,68]. These transcriptomic studies provide a foundation for the identification of novel regulators associated with faba bean-pathogen interactions and also a valuable source of markers for molecular breeding applications in this crop.

Translation of genomic resources from the model species *M. truncatula* or other sequenced related legume species such as chickpea, should be further exploited to raise the prospects in molecular faba bean breeding programs. The availability of large sets of conserved ESTs from model or related species constitutes a valuable source of markers that are physically associated with coding regions. These are good candidates for gene cloning or faba bean marker assisted selection. This is the approach used in this study to integrate all the faba bean genomic information so far reported, and to generate a new tool of reference for faba bean breeding and genomics approaches.

## Conclusions

We have constructed the first marker consensus genetic linkage map for faba bean by integrating segregation data from three recombinant inbred line populations, together with new common markers genotyped in this study. The final integrated map has allowed to (i) join a larger number of markers than in any previous individual map, (ii) obtain a more complete coverage of the faba bean genome, (iii) fill a number of gaps in previous independent maps, and (iv) improve the resolution of key QTLs. The colinearity of the consensus map was well maintained and will serve as reference for future faba bean multiple-line cross QTL mapping studies. Since 60% of the markers in the most developed map (RIL1) corresponded to coding regions, this consensus enhanced-density faba bean map provides a functional framework for candidate gene studies, expression analysis, comparative genomics, evolution studies and anchoring of the future faba bean genome sequences.

## Methods

### Mapping populations

The most recent maps of three RIL mapping populations,Vf6 × Vf27 (RIL1), Vf6 × Vf136 (RIL 2) and 29H × Vf136 (RIL3), were used to develop an integrated map of faba bean (Table 1). Vf6 was a common female parent in two progenies, Vf6 × VF136, which segregates for broomrape and *Ascochyta* resistance [7,8,24-26] and Vf6 × VF27, first reported by [6] and further used to construct the first exclusively gene-based genetic map in the species [11] and to identify and validate QTLs controlling flowering time and other yield-related traits [13]. Vf136 was the common male parent with the third population, 29H × Vf136, segregating for resistance to broomrape and *A. fabae *[10,15]. The populations consisted of 124 RILs for RIL1, 165 individual lines for RIL2, and 119 for RIL3.

The female parents, Vf6 and 29H are equina medium-seeded field beans with beige seed coat and resistance to *A. fabae*, Vf136 is also an equina type with reported resistance to broomrape and Vf27 is a black and small-seeded paucijuga form, supposedly close to a putative wild faba bean progenitor [69].

### Marker analyses

Genomic DNA was extracted from young leaves using liquid nitrogen and the procedure was as described by [3]. To increase marker density and to provide common markers to anchor the LGs from different populations, new markers from different sources were assayed. A set of SSRs, resistant gene analogs (RGAs), defence delated (DR) genes and ITAPs designed from different legume ESTs (*M. truncatula*, pea, lentil, lupin and soybean), were tested in the parental lines and the polymorphic ones genotyped in the corresponding RIL population.

### SSR markers

Fifty four faba bean SSRs [70] and 41 pea SSRs [71], were assayed for polymorphism among the parental lines using their respective protocols. In case of pea SSRs the reaction mixture was modified slightly by using 2,5 mM of MgCl_2_ and 1U Taq polymerase instead, to facilitate the orthologous amplification. SSRs revealing consistent and easily scorable bands were genotyped in the whole populations after electrophoresis in 2.5% - 3% agarose gels.

### RGAs and DR genes

Ten RGA classes were tested using PCR conditions described by [72]. To reveal polymorphism, amplification products for each RGA class were digested with a set of restriction enzymes according to the manufacturer’s instructions to obtain CAPS (Cleaved Amplified Polymorphic Sequences). Twelve additional RGAs [73,74] along with 12 DR genes, cloned and mapped in different legume species and mapped in pea [74], were also assayed. Amplifications and PCR conditions were as described by [72].

### Intron-targeted amplified polymorphic markers (ITAPs)

A total of 635 EST derived markers developed within the Grain Legumes Integrated Project (GLIP-Food-CT-2004-506223), were tested for polymorphism among the parental lines using the amplification protocols reported by [13,26]. Special efforts were focused on genotyping the ITAPs previously mapped in the most advanced Vf6 × Vf27 map [11,13]. As mentioned above, when no polymorphism was detected on agarose gels, PCR products amplified from both parents were digested with a range of restriction endonucleases in order to detect a SNP as a CAPS (Cleaved Amplified Polymorphic Sequence) marker that was further genotyped in the corresponding population.

### Saturation mapping

In order to saturate targeted regions conferring broomrape or ascochyta blight resistance we applied the BSA [75] based on previous QTL mapping information in cross Vf6 × Vf136 [24,26]. BSA has been widely adopted as a method to rapidly identify molecular makers in specific genome regions. The BSA principle consists in pooling DNAs from individuals from a segregating population according to two phenotypic classes. The resulting DNA bulks are equivalent to those from two Near Isogenic Lines (NILs) for which is assumed to generate a random genetic background at all other unlinked loci. In this study segregating individuals were grouped according to the genotype of markers flanking already localized QTLs. The contrasting pools were then screened with new markers in order to identify recombinants within each QTL interval.

QTLs underlying resistance to *O. crenata* and *A. fabae* were named *Oc* and *Af,* respectively [24,26]. Accordingly, bulks of plants fixed for alleles of the two markers flanking four QTL regions were selected from the RIL population: OPN07_1409_ and OPAI13_1018_ (flanking *Oc2* on linkage group VI.B), OPC19_1059_ and OPD12_425_ (*Oc3* on LG II.A), OPF08_710_ and OPW15_576_ (*Af1* on chr. III), OPAG5_737_ and MER02_1469_ (*Af2* on chr. II). A total of 748 RAPD primers was used in search for polymorphisms between the two bulks. For a given target region, markers showing expected differences between the pair of bulks were subsequently used to screen 14 plants individually. Markers that maintained the expected pattern of polymorphisms, were then used to screen the entire RIL population.

### Quantitative traits

Traits and QTL information were selected from seven published works [7,8,10,13,15,24,26] and supplemented by the bulked segregant analysis (in cross VF6 × VF136) and the saturation process described above. Trait descriptions, evaluation methods and abbreviations were assigned according to the previous references. Thus, the nine traits considered (Figure 1; Additional file 2), were the following: (1) *Orobanche crenata* resistance (trait abbreviation OC); (2) *Orobanche foetida* resistance (OF); (3) *Ascochyta fabae* resistance: disease severity on leaves (DSL); (4) *Ascochyta faba* resistance: disease severity on stems (DSS); (5) Days to flowering (DF); (6) Flowering length (FL); (7) Pod length (PL); (8) Number of ovules per pod (NOP); (9) Number of seeds per pod (NSP). Most of these QTLs showed to be stable as were identified and validated in different environments or genetic background. Each QTL was treated independently, making it possible to notice the number of times that a QTL is reported in a similar genomic location across independent experiments (Figure 1; Additional file 2).

### Data analysis

#### *Map construction and QTL analysis in Vf6 × VF136 (RIL 2)*

MAPMAKER 3.0 [76] was used to identify linkage groups using an LOD score of four as the threshold for considering significant linkage. MSTMap software [77] was used to determine maker orders by finding the minimum spanning tree of a graph for each linkage group. MAPMAKER was used to confirm marker orders determined by MSTMap and to convert the recombination fractions to centiMorgans (cM) using the mapping function of Kosambi [78].

QTL analysis was conducted using composite interval mapping (CIM) and multiple interval mapping (MIM) in Windows QTL Cartographer V2.5 [79]. Markers to be used as cofactors for CIM were selected by forward-backward stepwise regression. The number of markers controlling the genetic background in CIM was set to five. The thresholds for the detection of QTLs were estimated by permutations analysis [80] using 1,000 permutations.

### Consensus map construction

Three individual genetic maps were used to generate a consensus map using MergeMap [42] by converting the individual maps into directed acyclic graphs (DAGs) that are then merged in consensus graph on the basis of their shared vertices [81]. As MergeMap tends to inflate genetic distances in the consensus genetic map [42,82], marker data from different mapping populations were pooled together and the order of each consensus linkage group as established by MergeMap was set, in order to calculate genetic distances using MAPMAKER. The consensus map for each linkage group was visualized by MapChart [83].

## Competing interests

The authors declare that they have no competing interests.

## Authors’ contributions

ZS performed the statistical analyses for map merging, integrated QTL information, prepared tables and graphic representations and helped to draft the manuscript. CMA generated marker and QTL data for the mapping populations, participated in the design of the study and assisted with manuscript preparation and editing. SCI, RDR performed additional marker genotyping and provided new QTL data. GGR, CP, NG, SV, SOM and MVG provided new marker data for mapping saturation and anchoring points for map integration. JIC assisted with manuscript editing. AMT coordinated the map integration study, contribute to the analysis and interpretation of data, provided the marker and QTL data and drafted the manuscript. All authors read and approved the final manuscript.

## Supplementary Material

Additional file 1: Table S1 **(A)** Putative QTLs for flowering time and yield related traits detected in the faba bean RIL population Vf6 × Vf27 (from Cruz-Izquierdo et al., 2012 with modifications). **(B)** Putative QTLs for *Ascochyta fabae*, *Orobanche crenata* and *Orobanche foetida* resistance detected in the faba bean RIL population Vf6 × Vf136 (Díaz-Ruíz et al., 2009a; 2009b; 2010 and this study). **(C)** Putative QTLs for *Orobanche crenata* and *Orobanche foetida* resistance detected in the faba bean RIL population 29H × Vf136 (from Gutierrez et al., 2013 with modifications). Click here for file

Additional file 2: Table S2 Information on the markers used in this study and mapped in the three faba bean RIL populations. Click here for file
