# Supplementary material for: A reference consensus genetic map for molecular markers and economically important traits in faba bean (Vicia faba L.)
Source: BMC Genomics. 2013 Dec 30;14:932. doi: 10.1186/1471-2164-14-932 (PMC3880837; doi:10.1186/1471-2164-14-932)
Supplement: Additional file 1: Table S1 — (A) Putative QTLs for flowering time and yield related traits detected in the faba bean RIL population Vf6 × Vf27 (from Cruz-Izquierdo et al., 2012 with modifications). (B) Putative QTLs for Ascochyta fabae, Orobanche crenata and Orobanche foetida resistance detected in the faba bean RIL population Vf6 × Vf136 (Díaz-Ruíz et al., 2009a; 2009b; 2010 and this study). (C) Putative QTLs for Orobanche crenata and Orobanche foetida resistance detected in the faba bean RIL population 29H × Vf136 (from Gutierrez et al., 2013 with modifications). [file 1471-2164-14-932-S1.doc]

Supplementary Table S1:

(A) Putative QTLs for flowering time and yield related traits detected in the faba bean RIL population Vf6 × Vf27 (from Cruz-Izquierdo et al., 2012 with modifications)

| Trait | Locality | Year | QTL | Chromosome / LG | Flanking markers | LOD | Add | R^2^ |
| --- | --- | --- | --- | --- | --- | --- | --- | --- |
| DF | Córdoba | 2007 | *df-2007-1* | V | AnMtS37 | 11.75 | 5.260 | 25.33 |
| DF | Córdoba | 2007 | *df-2007-2* | IV | 1947_02 | 5.11 | -2.640 | 9.41 |
| DF | Córdoba | 2007 | *df-2007-3* | I | OPI11_1453 | 4.43 | -2.430 | 8.07 |
| DF | Córdoba | 2007 | *df-2007-4* | IV | CNGC4 / PPH | 3.11 | 2.140 | 6.29 |
| DF | Córdoba | 2007 | *df-2007-5* | III | Lup328 / cgP137F | 3.04 | 2.490 | 8.65 |
| DF | Córdoba | 2008 | *df-2008-1* | V | Pis_GEN_6_3_1 | 7.77 | 5.530 | 21.95 |
| DF | Córdoba | 2008 | *df-2008-2* | I | mtmt_GEN_01102_02_1 | 3.52 | -2.730 | 8.50 |
| FL | Córdoba | 2007 | *fl-2007-1* | V | Prx-1 / Pis_GEN_6_3_1 | 7.31 | -5.300 | 28.78 |
| FL | Córdoba | 2008 | *fl-2008-1* | V | Pis_GEN_6_3_1 | 5.07 | -4.180 | 13.69 |
| FL | Córdoba | 2008 | *fl-2008-2* | I | mtmt_GEN_01102_02_1 | 4.43 | 2.930 | 11.01 |
| PL | Córdoba | 2007 | *pl-2007-1* | V | GLIP099 | 7.53 | -0.580 | 23.53 |
| PL | Córdoba | 2007 | *pl-2007-2* | I | mtmt_GEN_00447_04_3 | 5.81 | 0.320 | 15.32 |
| PL | Córdoba | 2007 | *pl-2007-3* | IIa | OPA11_756 / LG34b | 4.48 | 0.270 | 11.43 |
| PL | Córdoba | 2007 | *pl-2007-4* | IV | PRAT | 4.14 | 0.260 | 10.45 |
| PL | Córdoba | 2008 | *pl-2008-1* | V | GLIP099 | 4.30 | -0.520 | 13.92 |
| PL | Córdoba | 2008 | *pl-2008-2* | IIa | Lup242 | 3.84 | 0.320 | 10.53 |
| NOP | Córdoba | 2007 | *nop-2007-1* | VI | Lup280 | 8.86 | -0.200 | 26.78 |
| NOP | Córdoba | 2008 | *nop-2008-1* | VI | Lup280 | 3.81 | -0.140 | 11.36 |
| NSP | Córdoba | 2007 | *nsp-2007-1* | VI | OPA11_664 / CP450 | 8.17 | -0.210 | 25.22 |
| NSP | Córdoba | 2007 | *nsp-2007-2* | I | GLIP071 / mtmt_GEN_00447_04_3 | 4.71 | 0.190 | 17.98 |
| NSP | Córdoba | 2007 | *nsp-2007-3* | I | CHR | 3.41 | -0.150 | 9.95 |
| NSP | Córdoba | 2007 | *nsp-2007-4* | II | OPL18_751 / OPA19_865 | 3.39 | 0.140 | 11.51 |
| NSP | Córdoba | 2008 | *nsp-2008-1* | VI | OPA11_664 / CP450 | 8.52 | -0.270 | 37.93 |

Traits: DF - days to flowering; FL - flowering length; PL - pod length; NOP - number of ovules per pod; NSP - number of seeds per pod

QTL - quantitative trait locus; LOD - maximum LOD value; Add - additive effect; R^2^ - proportion of phenotypic variance explained by the respective QTL (%)

(B) Putative QTLs for *Ascochyta fabae*, *Orobanche* *crenata* and *Orobanche* *foetida* resistance detected in the faba bean RIL population Vf6 × Vf136 (Díaz-Ruíz et al., 2009a; 2009b; 2010 and this study)

| Trait | Locality | Year | QTL | Chromosome / LG | Flanking markers* | LOD | Add | R^2^ |
| --- | --- | --- | --- | --- | --- | --- | --- | --- |
| DSL | Córdoba | 2003 | *Af1_DSL* | III | Lup066 / OPZ08_530 | 4.888 | -1.190 | 11.74 |
| DSL | Córdoba | 2003 | *Af2_DSL* | II | Pis_GEN_23_5_6_1 / Mer04_790 | 4.998 | -1.179 | 12.54 |
| DSS | Córdoba | 2003 | *Af1_DSS* | III | Lup066 / OPZ08_530 | 3.569 | -3.018 | 8.76 |
| DSS | Córdoba | 2003 | *Af2_DSS* | II | OPE17_1326 / OPD12_425 | 3.575 | -3.280 | 9.85 |
| OC | Córdoba | 2003 | *Oc6_C3* | I | OPG07_1714 / OPH01_900 | 2.49 | -0.055 | 7.74 |
| OC | Córdoba | 2003 | *Oc3_C3* | II | OPM15_794 / Pis_GEN_4_3_1 | 2.07 | -0.050 | 6.52 |
| OC | Córdoba | 2003 | *Oc14_C3*** | II | OPP10_555 | 2.71 | -0.049 | 6.08 |
| OC | Córdoba | 2003 | *Oc2_C3* | VI | OPAG11_956 / OPI05_1019 | 6.49 | -0.090 | 19.82 |
| OC | Córdoba | 2004 | *Oc4_C4* | I | OPB03_289 | 4.15 | -0.061 | 9.77 |
| OC | Córdoba | 2004 | *Oc3_C4* | II | OPM15_794 / Pis_GEN_4_3_1 | 2.34 | -0.057 | 8.40 |
| OC | Córdoba | 2004 | *Oc16_C4*** | V | OPAD02_1282 / OPK18_1049 | 2.37 | -0.046 | 5.66 |
| OC | Córdoba | 2004 | *Oc2_C4* | VI | OPAG11_956 / OPI05_1019 | 3.31 | -0.067 | 11.47 |
| OC | Mengíbar | 2004 | *Oc5_M4* | I | OPM18_1620 / OPA17_524 | 2.63 | -0.053 | 9.42 |
| OC | Mengíbar | 2004 | *Oc15_M4*** | II | OPAF20_776b / Pis_GEN_58_3_4_1 | 2.66 | -0.049 | 7.90 |
| OC | Mengíbar | 2004 | *Oc2_M4* | VI | OPAI13_1018 / OPAC06_396 | 2.46 | -0.048 | 7.50 |
| OF | Beja | 2003 | *Of1_B3* | I | OPAH13_475 / OPM18_1689 | 2.33 | -0.046 | 7.30 |
| OF | Beja | 2004 | *Of2_B4* | III | Pis_GEN_25_2_3_1 / B3 | 4.35 | -0.087 | 16.38 |

Traits: DSL - *Ascochyta* *fabae* resistance: disease severity on leaves; DSS - *Ascochyta* *fabae* resistance: disease severity on stems; OC - *Orobanche* *crenata* resistance; OF - *Orobanche foetida* resistance

QTL - quantitative trait locus; LOD - maximum LOD value; Add - additive effect; R^2^ - proportion of phenotypic variance explained by the respective QTL (%)

*Underlined markers were added to the previous map after BSA analysis

**Novel QTLs detected in this study

(C) Putative QTLs for *Orobanche* *crenata* and *Orobanche* *foetida* resistance detected in the faba bean RIL population 29H × Vf136 (from Gutierrez et al., 2013 with modifications)

| Trait | Locality | Year | QTL | Chromosome / LG | Flanking markers | LOD | Add | R^2^ |
| --- | --- | --- | --- | --- | --- | --- | --- | --- |
| OC | Córdoba | 2006 | *Oc7_C6* | VI | OPU09_831 / OPI10_1178 | 9.44 | -0.090 | 33.31 |
| OC | Córdoba | 2006 | *Oc8_C6* | V | OPAC06_1171 / mtmt_GEN_00866_02_1 | 3.29 | -0.050 | 9.81 |
| OC | Córdoba | 2007 | *Oc7_C7* | VI | OPU09_831 / OPA17_633 | 6.69 | -0.070 | 22.80 |
| OC | Córdoba | 2007 | *Oc8_C7* | V | mtmt_GEN_00866_02_1 / OPAC06_1034 | 3.75 | -0.050 | 13.20 |
| OC | Córdoba | 2008 | *Oc7_C8* | VI | OPU09_831 / OPA17_633 | 8.85 | -0.090 | 29.92 |
| OC | Córdoba | 2008 | *Oc9_C8* | LG02 | OPG16_908 / OPU11_1232 | 3.29 | -0.050 | 10.03 |
| OC | Kafr El-Sheikh | 2007 | *Oc10_K7* | III | OPD20_322 / OPB19_644 | 2.80 | 0.060 | 11.80 |
| OC | Kafr El-Sheikh | 2007 | *Oc11_K7* | LG26 | OPG04_810 / OPQ19_1605 | 2.12 | -0.060 | 10.72 |
| OC | Kafr El-Sheikh | 2007 | *Oc12_K7* | I | OPJ18_858 / Pis_GEN_14_7_1 | 2.15 | -0.050 | 7.96 |
| OC | Kafr El-Sheikh | 2007 | *Oc13_K7* | LG08 | OPJ01_896 / OPD10_1185 | 2.22 | 0.060 | 13.01 |
| OF | Beja | 2006 | *Of3_B6* | V | Vfg69 / OPAC06_1034 | 3.43 | -0.060 | 12.34 |
| OF | Beja | 2006 | *Of4_B6* | II | TBB2 | 3.83 | 0.300 | 12.98 |
| OF | Beja | 2006 | *Of5_B6* | IIa | RGA01 / OPW15_924 | 3.84 | 0.390 | 18.74 |

Traits: OC - *Orobanche* *crenata* resistance; OF - *Orobanche foetida* resistance

QTL - quantitative trait locus; LOD - maximum LOD value; Add - additive effect; R^2^ - proportion of phenotypic variance explained by the respective QTL (%)
